# Differential Circulating Levels of Naturally Occurring Antibody to α-Synuclein in Parkinson’s Disease Dementia, Alzheimer’s Disease, and Vascular Dementia

**DOI:** 10.3389/fnagi.2020.571437

**Published:** 2020-09-25

**Authors:** Jian Wang, Bo Zheng, Shu Yang, Mei Hu, Jian-Hong Wang

**Affiliations:** ^1^Department of Neurology, Yaan People’s Hospital, Yaan, China; ^2^Department of Neurology, Sichuan Provincial People’s Hospital, University of Electronic Science and Technology of China, Chengdu, China; ^3^Department of Imaging, Sichuan Provincial People’s Hospital, University of Electronic Science and Technology of China, Chengdu, China

**Keywords:** α-Synuclein, dementia, naturally occurring antibody, biomarker, circulating

## Abstract

**Background**: Aggregation of alpha-synuclein (α-Syn) is considered to be a significant pathological hallmark and a driving force of Parkinson’s disease (PD). PD dementia (PDD) occurs in a substantial number of PD patients. Naturally occurring antibody against α-Syn (NAb-α-Syn) exists ubiquitously in human blood and is reported to be altered in PD. However, it is not clear yet whether PDD had similar changes of circulating NAb-α-Syn.

**Methods**: In this study, we recruited 61 PDD patients, 52 patients with Alzheimer’s disease (AD), 51 patients with vascular dementia (VaD), and 50 normal controls (NCs). ELISA was used to examine NAb-α-Syn levels in serum.

**Results**: In comparison with NCs, serum levels of NAb-α-Syn were significantly lower in patients with PDD. However, serum levels of NAb-α-Syn were comparable among AD, VaD, and NC groups. Serum levels of NAb-α-Syn were positively correlated with the cognitive function, as reflected by Mini-Mental State Examination (MMSE) and Montreal Cognitive Assessment (MoCA). Serum levels of NAb-α-Syn were negatively correlated with the severity of PD [as reflected by the Unified Parkinson Disease Rating Scale (UPDRS)] and the duration of PD and PDD. Serum NAb-α-Syn can differentiate PDD patients from AD and VaD patients.

**Conclusion**: These results suggest that circulating NAb-α-Syn might be a potential biomarker of PDD.

## Introduction

Neurodegenerative disorders such as Parkinson’s disease (PD), Alzheimer’s disease (AD), and Creutzfeldt–Jakob disease (CJD) are characterized by the deposition and aggregation of misfolded proteins in the central nervous system (CNS; Skovronsky et al., 2006). The characteristic pathological changes in PD consist of dopaminergic neuronal loss, gliosis, and intraneuronal Lewy body formation in the substantia nigra pars compacta and striatum. Alpha-Synuclein (α-Syn) was found to be the predominant protein in Lewy body inclusions (Tofaris and Spillantini, 2007).

Although PD is considered to be a motor disorder, non-motor symptoms may occur from early stages, even before the manifestation of motor symptoms. Dementia can occur in a substantial number of PD patients with a prevalence of about 30% (Hanagasi et al., 2017). PD dementia (PDD) is characterized by a dysexecutive syndrome with early and prominent impairment of attention and visuospatial functions, moderately impaired episodic memory, and relatively preserved core language functions (Hanagasi et al., 2017). Although the symptoms and clinical courses vary in PDD and other types of dementia, there is some overlap of clinical characteristics among different dementias. Currently, blood-based biomarkers are limited to distinguish them. After damage of neurons or axons, soluble α-Syn is released into the cerebral spinal fluid and efflux to the circulation in PD patients and in healthy individuals (El-Agnaf et al., 2006). This event may stimulate an induction of autoantibody formation against α-Syn [naturally occurring antibody against α-Syn (NAb-α-Syn); Papachroni et al., 2007]. The levels of circulating NAb-α-Syn have been reported to be altered in PD patients (Besong-Agbo et al., 2013). However, studies investigating the levels of NAb-α-Syn in PDD patients are limited. In this study, we investigated whether circulating NAb-α-Syn was capable of distinguishing PDD from AD and vascular dementia (VaD).

## Materials and Methods

### Participants

A total number of 61 PDD, 52 AD, and 51 VaD patients were consecutively recruited from the Department of Neurology, Sichuan Provincial People’s Hospital. Fifty normal controls (NCs) without cognitive dysfunction were recruited from the health examination center of Sichuan Provincial People’s Hospital. PDD was diagnosed according to criteria proposed by the Movement Disorder Society Task Force (Emre et al., 2007). Patients who developed dementia within 1 year after PD onset were excluded. AD diagnoses were based on the clinical criteria for probable AD as described by the National Institute on Aging-Alzheimer’s Association (McKhann et al., 2011). The diagnosis of VaD was based on the Diagnostic and Statistical Manual of Mental Disorders–fourth Edition (DSM-IV) and also fulfilled the imaging criteria for VaD (Erkinjuntti et al., 2000). Patients with cognitive and behavioral symptoms presenting as a result of other conditions, such as acute confusion due to systemic disease, abnormality or drug intoxication, depression, normal pressure hydrocephalus, progressive supranuclear palsy, or history of significant brain trauma followed by persistent neurologic deficit or known structural brain abnormality, were excluded. All participants received cognitive assessment using Mini-Mental State Examination (MMSE) and Montreal Cognitive Assessment (MoCA). Severity of PD was assessed by the Unified Parkinson Disease Rating Scale (UPDRS). Informed consent was obtained from all patients and their legal relatives. This research project was approved by the institutional review boards at Sichuan Provincial People’s Hospital.

### Isolation of Naturally Occurring Antibody Against Alpha-Synuclein

NAb-α-Syn isolated from human intravenous immunoglobulin G (IVIg; Yuanda, Sichuan, China) was used as a standard. NAb-α-Syn was extracted using affinity chromatography. Briefly, a column was packed with 2 ml resin (PIERCE Biotechnology, Rockford, IL, USA), labeled with 1 mg recombinant α-Syn (rPeptide, Bogart, GA, USA), equilibrated, and washed with phosphate-buffered saline (PBS, pH 7.4). After passing purified IVIg through the column, fractions were eluted with glycine buffer, pH 2.8. The main fractions containing the highest amount of NAb-α-Syn were pooled, and their concentration was determined using the NanoDrop spectrometer (Nanodrop1000, PeqLab, Erlangen, Germany). Pooled NAb-α-Syn was stored at −20°C until use.

### Naturally Occurring Antibody Against Alpha-Synuclein ELISA

High-bind 96-well ELISA plates (Nunc, Denmark) were coated overnight with recombinant α-Syn 50 μg/ml (100 ml/well) in phosphate-coating buffer (1.7 mM sodium dihydrogen phosphate, 98 mM disodium hydrogen phosphate, 0.05% sodium azide; pH 7.6) at 4°C. Wells were then blocked with 5% bovine serum albumin (BSA; Sigma, St. Louis, MO, USA) in PBS for 1 h at 37°C. Standards were prepared by diluting purified NAb-α-Syn from IVIg in dilution buffer (5% BSA). Sera were diluted 1:100. Plates were washed four times with 300 ml washing buffer (PBS with 0.05% Tween-20) and incubated with standards and samples (100 ml/well) for 1 h at room temperature (RT). After washing, plates were incubated with 100 ml/well of detection antibody, a 1:5,000 dilution of peroxidase-labeled goat antihuman IgG antibody (Sigma, St. Louis, MO, USA) in dilution buffer, at RT for 1 h. After a final wash, the assay was developed using 100 ml/well TMB (Sigma, St. Louis, MO, USA) for 15 min. The reaction was stopped with 30 ml 2 N sulfuric acid (Roth, Karlsruhe, Germany) and read at 450 nm. The difference in signal between coated and uncoated wells was considered to be solely due to α-Syn-nAbs binding to α-Syn and was used for further calculations.

### Statistics

Statistical analysis was performed using SPSS (version 19.0, SPSS, Chicago, IL, USA). The Kolmogorov–Smirnov and Shapiro–Wilk tests were used to check for normal distribution. To compare demographic, clinical, and serum data between groups, parametric and nonparametric tests such as one-way analysis of variance were used. Kruskal–Wallis test was used to compare multiple groups (PDD, AD, VaD, and NC), followed by *post hoc* pairwise comparisons using Dunn multiple comparison procedures. In order to test for significant correlations between clinical and demographic characteristics of patients and the serum data, Spearman rank correlation was used. Optimal sensitivity and specificity were determined *via* the receiver operating characteristic (ROC) curve analysis to determine the capacity of NAb-α-Syn in differentiating different types of dementia. A *p* < 0.05 was regarded as significant.

## Results

### Demographics

The present study recruited 61 PDD patients, 52 AD patients, 51 VaD patients, and 50 NCs. The average age was not significantly different among groups (NC: 66.68 ± 8.63 years, PDD: 69.07 ± 8.25 years, AD: 66.90 ± 9.58 years, VaD: 65.00 ± 8.99 years; *p* = 0.115). The percentages of females were similar among groups (NC: 50.00%, PDD: 50.82%, AD: 48.08%, VaD: 47.06%; *p* = 0.978). However, the percentage of ApoE ε4 carriers was significantly higher in the AD group (NC: 20.00%, PDD: 19.67%, AD: 46.15%, VaD: 19.61%; *p* = 0.003). The average education level was also not significantly different among groups (NC: 8.84 ± 4.49 years, PDD: 8.92 ± 4.55 years, AD: 8.08 ± 4.27 years, VaD: 8.47 ± 4.58 years; *p* = 0.752). The cognitive status, as reflected by MMSE (NC: 27.96 ± 1.88, PDD: 14.52 ± 6.62, AD: 14.87 ± 6.21, VaD: 15.27 ± 5.37; *p* < 0.001) and MoCA (NC: 28.12 ± 1.76, PDD: 12.90 ± 6.24, AD: 13.19 ± 5.15, VaD: 12.10 ± 4.61; *p* < 0.001), was significantly different among groups (Table 1).

**Table 1:** Demographic data of subjects.

	NC (*n* = 50)	PDD (*n* = 61)	AD (*n* = 52)	VaD (*n* = 51)	*p*
Age, years	66.68 ± 8.63	69.07 ± 8.25	66.90 ± 9.58	65.00 ± 8.99	0.115
Female, n (%)	25 (50.00)	31 (50.82)	25 (48.08)	24 (47.06)	0.978
ApoE ε4, n (%)	10 (20.00)	12 (19.67)	24 (46.15)	10 (19.61)	0.003
Education, years	8.84 ± 4.49	8.92 ± 4.55	8.08 ± 4.27	8.47 ± 4.58	0.752
MMSE	27.96 ± 1.88	14.52 ± 6.62	14.87 ± 6.21	15.27 ± 5.37	<0.001
MoCA	28.12 ± 1.76	12.90 ± 6.24	13.19 ± 5.15	12.10 ± 4.61	<0.001
UPDRS	NA	29.70 ± 1.75	NA	NA	NA
Duration of PD	NA	7.26 ± 3.83	NA	NA	NA
Duration of dementia	NA	2.57 ± 2.51	5.73 ± 2.96	6.04 ± 3.38	<0.001

*Age, education, MMSE, MoCA, and UPDRS were expressed as means ± SD. Female and ApoE ɛ4 carriers were expressed as number (frequencies). NA, not applicable; NC, normal control; PDD, Parkinson’s disease dementia; AD, Alzheimer’s disease; VaD, vascular dementia; MMSE, Mini-Mental State Examination; MoCA, Montreal Cognitive Assessment; UPDRS, Unified Parkinson Disease Rating Scale*.

### Serum Naturally Occurring Antibody Against Alpha-Synuclein Levels in Different Types of Dementia

**Figure 1:**
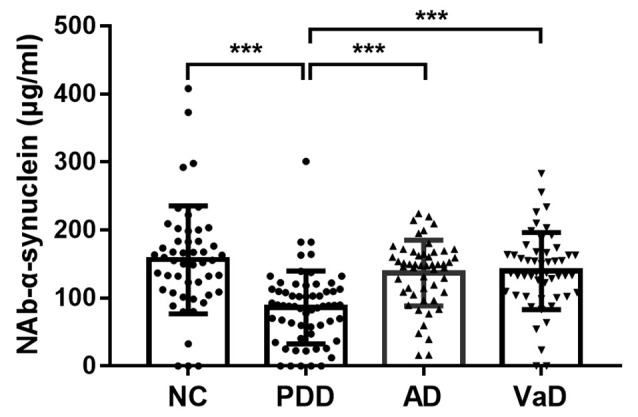
Serum levels of naturally occurring antibody against alpha-synuclein (NAb-α-Syn) in different types of dementia. Serum levels of NAb-α-Syn were significantly higher in the Parkinson’s disease dementia (PDD) group in comparison with those in the normal control (NC), Alzheimer’s disease (AD), and vascular dementia (VaD) groups. ***p < 0.001. One-way ANOVA.

We first investigated serum levels of NAb-α-Syn in different types of dementia. We found that serum levels NAb-α-Syn were significantly higher in the PDD group (86.2 ± 53.6 pg/ml) in comparison with those in the NC (156.3 ± 79.4 pg/ml; *p* < 0.001), AD (136.9 ± 48.2 pg/ml; *p* < 0.001), and VaD (139.7 ± 56.6 pg/ml; *p* < 0.001) groups. However, no significant difference was found among the NC, AD, and VaD groups (Figure 1). These findings indicated that the alteration of serum NAb-α-Syn levels was relatively specific in PDD.

### Correlations of Serum Naturally Occurring Antibody Against Alpha-Synuclein Levels With Cognitive Function

**Figure 2:**
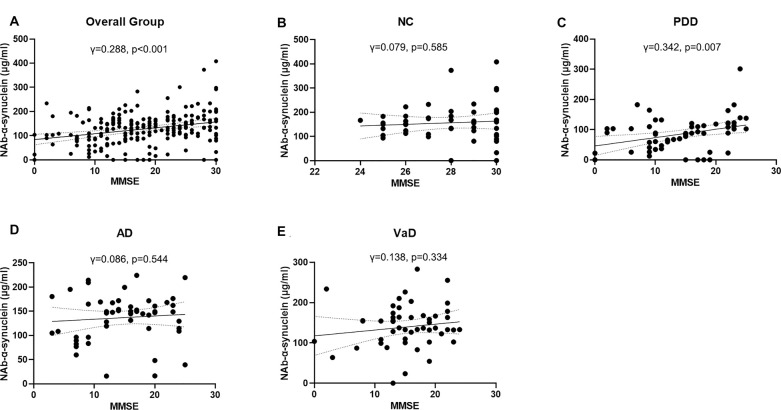
Correlations between serum levels of naturally occurring antibody against alpha-synuclein (NAb-α-Syn) and Mini-Mental State Examination (MMSE). Correlations between serum levels of NAb-α-Syn in the **(A)** overall group, **(B)** normal control (NC) group, **(C)** PDD group, **(D)** AD group, and **(E)** VaD group. Spearman correlation analysis.

**Figure 3:**
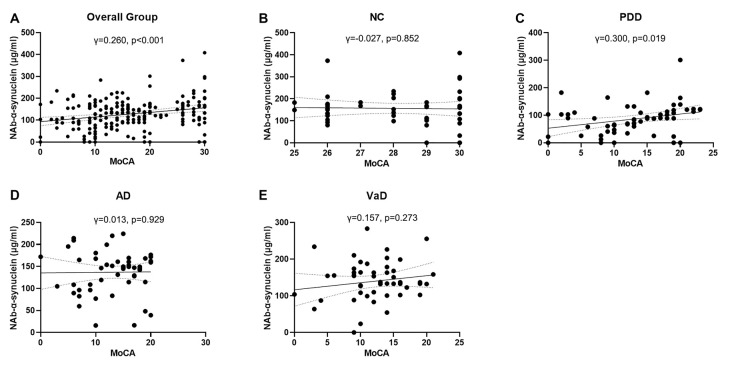
Correlations between serum levels of naturally occurring antibody against alpha-synuclein (NAb-α-Syn) and Montreal Cognitive Assessment (MoCA). Correlations between serum levels of NAb-α-Syn in the **(A)** overall group, **(B)** normal control (NC) group, **(C)** PDD group, **(D)** AD group, and **(E)** VaD group. Spearman correlation analysis.

We next investigated the correlations of serum NAb-α-Syn levels with the cognitive functions. We found that serum NAb-α-Syn levels were significantly correlated with MMSE in the overall group (*γ* = 0.288, *p* < 0.001). Subgroup analysis indicated that serum NAb-α-Syn levels were significantly correlated with MMSE in the PDD group (*γ* = 0.342, *p* = 0.007), but not in the NC (*γ* = 0.079, *p* = 0.585), AD (*γ* = 0.086, *p* = 0.544), or VaD (*γ* = 0.138, *p* = 0.334) group (Figure 2). Similarly, serum NAb-α-Syn levels were significantly correlated with MoCA in the overall group (*γ* = 0.260, *p* < 0.001) and the PDD subgroup (*γ* = 0.300, *p* = 0.019), but not in the NC (*γ* = −0.027, *p* = 0.852), AD (*γ* = 0.013, *p* = 0.929), or VaD group (*γ* = 0.157, *p* = 0.273; Figure 3).

### Correlations of Serum Naturally Occurring Antibody Against Alpha-Synuclein Levels With Disease Severity and Duration

**Figure 4:**
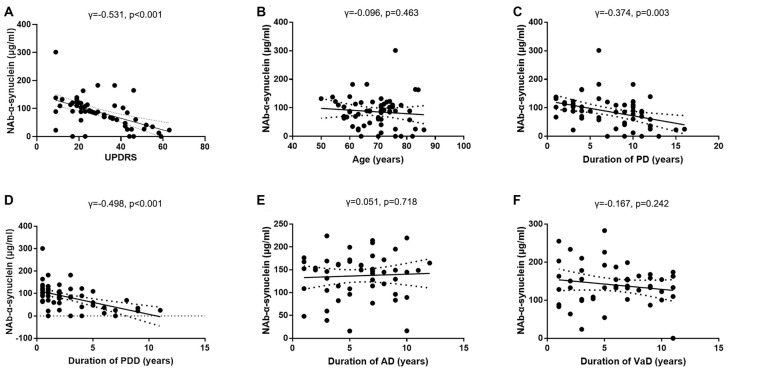
Correlations of serum levels of naturally occurring antibody against alpha-synuclein (NAb-α-Syn) with disease severity and duration. **(A)** Correlation between serum levels of NAb-α-Syn and Unified Parkinson Disease Rating Scale (UPDRS) of PDD. **(B)** Correlation between serum levels of NAb-α-Syn and age of PDD. **(C)** Correlation between serum levels of NAb-α-Syn and duration of Parkinson’s disease (PD). **(D)** Correlation between serum levels of NAb-α-Syn and duration of PDD. **(E)** Correlation between serum levels of NAb-α-Syn and duration of AD. **(F)** Correlation between serum levels of NAb-α-Syn and duration of VaD. Spearman correlation analysis.

We next investigated the correlations between serum NAb-α-Syn levels and UPDRS in the PDD group. We found that serum NAb-α-Syn levels were negatively correlated with UPDRS (*γ* = −0.531, *p* < 0.001; Figure 4A). However, serum NAb-α-Syn levels were not significantly correlated with the age of PDD patients (*γ* = −0.096, *p* = 0.463; Figure 4B). Serum NAb-α-Syn levels had a positive correlation with both the duration of PD (*γ* = −0.374, *p* = 0.003) and dementia (*γ* = −0.498, *p* < 0.001) in the PDD group (Figures 4C,D). However, serum NAb-α-Syn levels were not correlated with the duration of dementia in the AD (*γ* = 0.051, *p* = 0.718; Figure 4E) or VaD group (*γ* = −0.167, *p* = 0.242; Figure 4F).

### The Capacity of Serum Naturally Occurring Antibody Against Alpha-Synuclein in Differentiating Different Types of Dementia

**Figure 5:**
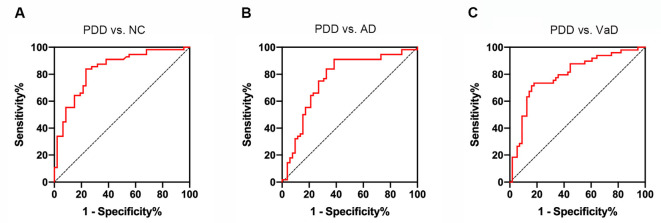
Receiver operating characteristic (ROC) curves of serum naturally occurring antibody against alpha-synuclein (NAb-α-Syn) in differentiating PDD from normal control (NC) and other types of dementia. **(A)** The ROC curve of serum NAb-α-Syn in differentiating PDD from NC. **(B)** The ROC curve of serum NAb-α-Syn in differentiating PDD from AD. **(C)** The ROC curve of serum NAb-α-Syn in differentiating PDD from VaD.

Using the ROC curve analysis, we found that the area under the ROC curve (AUC) of serum NAb-α-Syn in PDD vs. NC was 0.837 (*p* < 0.001, 95% confidence interval = 0.758–0.915). When applying the optimal cut-off value of 123.0 pg ml^−1^ calculated using the Youden index, the overall sensitivity and specificity of serum NAb-α-Syn for distinguishing PDD from NC was 83.9% and 76.6%, respectively (Figure 5A). The AUC of serum NAb-α-Syn in PDD vs. AD was 0.712 (*p* < 0.001, 95% confidence interval = 0.672–0.859). When applying the optimal cut-off value of 140.7 pg ml^−1^, the overall sensitivity and specificity of serum NAb-α-Syn for distinguishing PDD from AD were 91.1% and 61.5%, respectively (Figure 5B). The AUC of serum NAb-α-Syn in PDD vs. VaD was 0.794 (*p* < 0.001, 95% confidence interval = 0.706–0.882). When applying the optimal cut-off value of 140.7 pg ml^−1^, the overall sensitivity and specificity of serum NAb-α-Syn for distinguishing PDD from AD were 73.5% and 82.1%, respectively (Figure 5C).

## Discussion

In the present study, we found differential serum levels of NAb-α-Syn among PDD, AD, and VaD patients. Serum levels of NAb-α-Syn were correlated with the cognitive function and disease severity of PDD. Moreover, serum levels of NAb-α-Syn is a potential biomarker for differentiating PDD from AD and VaD.

α-Syn exists in its soluble form and can efflux from the brain into the periphery, which may stimulate the production of NAb-α-Syn. The levels of NAb-α-Syn in PD were controversial in previous studies (Besong-Agbo et al., 2013; Folke et al., 2019). Currently, few studies have investigated the circulating levels of NAb-α-Syn in PDD. We found in this study that circulating NAb-α-Syn was decreased in PDD patients. However, AD and VaD patients had comparable circulating NAb-α-Syn levels. These findings imply that the pattern of circulating NAb-α-Syn may have disease specificity among neurodegenerative diseases. The mechanism underlying the decrease of NAb-α-Syn is not clear yet. We found that serum NAb-α-Syn levels were correlated with the duration of PD and PDD, but not with the age of PDD patients, indicating that the alteration of NAb-α-Syn levels are not consequent to aging, but to the α-Syn pathogenesis. It is possible that NAb-α-Syn is consumed by increased circulating α-Syn.

The decrease of NAb-α-Syn in PDD may have some clinical and pathological relevance. We found that circulating levels of NAb-α-Syn were positively correlated with MMSE and MoCA scores in the overall group and PDD subgroup. Furthermore, circulating levels of NAb-α-Syn were also negatively correlated with UPDRS in the PDD group. These findings suggest an association between NAb-α-Syn and the severity of neurodegeneration in PDD. While the etiology of PDD is multifactorial, α-Syn is suggested to be a central component to the pathogenesis of the disease (Rocha et al., 2018). NAb-α-Syn may aid in the clearance of α-Syn, resulting in the alleviation of neuronal injury by α-Syn. This hypothesis is supported by previous studies, which suggests that NAb-α-Syn rescues memory and motor functions and attenuates α-Syn pathologies in animal models of PD (Huang et al., 2019). In this regard, NAb-α-Syn might be a physiological protective factor against α-Syn pathologies. The decrease of NAb-α-Syn in PDD might contribute to α-Syn-induced memory loss and motor dysfunctions.

Currently, the differential diagnosis of dementia is largely based on the symptom spectrum of patients. Recent progress in molecular imaging (Janelidze et al., 2020; Kantarci et al., 2020; Kozlova et al., 2020) and cerebrospinal fluid (CSF) biomarkers (Altomare et al., 2019; Llibre-Guerra et al., 2019) improved the diagnostic accuracy of dementia. However, due to the high cost and invasiveness of these diagnostic strategies, blood-based biomarkers are in urgent need for the diagnosis and differential diagnosis of dementia. Our ROC curve analysis found that circulating NAb-α-Syn has relatively high accuracy to differentiate PDD from AD and VaD. As there is some overlap of clinical manifestations among different types of dementia, NAb-α-Syn might be a potential differential diagnostic biomarker of PDD.

This study is limited by its cross-sectional nature and small sample size. Besides, this study is a pure correlative study that investigated the association between NAb-α-Syn and PDD. However, we found a disease-specific change of circulating NAb-α-Syn in PDD. This study also proposed a possible role of NAb-α-Syn in the pathogenesis of PDD from a clinical aspect.

## Data Availability Statement

The raw data supporting the conclusions of this article will be made available by the authors, without undue reservation.

## Ethics Statement

The studies involving human participants were reviewed and approved by Sichuan Provincial Hospital. The patients/participants provided their written informed consent to participate in this study.

## Author Contributions

J-HW and MH designed the study and drafted the manuscript. JW and BZ conducted the experiments, collected and analyzed the data, and drafted the manuscript. SY and MH were responsible for clinical assessment of the subjects.

## Conflict of Interest

The authors declare that the research was conducted in the absence of any commercial or financial relationships that could be construed as a potential conflict of interest.
